# Coronary Artery Circumferential Stress: Departure from Laplace Expectations with Aging

**DOI:** 10.1100/tsw.2009.109

**Published:** 2009-09-15

**Authors:** Richard E Tracy, Marsha L. Eigenbrodt

**Affiliations:** ^1^ Department of Pathology, Louisiana State University Health Sciences Center, New Orleans, USA; ^2^ Adjunct Department of Internal Medicine, University of Arkansas for Medical Sciences, Little Rock, USA

**Keywords:** aging, arteriosclerosis, atherosclerosis, human, intima-media thickness

## Abstract

Normal, youthful arteries generally maintain constant radius/wall thickness ratios, with the relationship being described by the Laplace Law. Whether this relationship is maintained during aging is unclear. This study first examines the Laplace relationships in postmortem coronary arteries using a novel method to correct measurements for postmortem artifacts, uses data from the literature to provide preliminary validation, and then describes histology associated with low circumferential stress. Measurements of radius and wall thickness, taken at sites free from atheromas, were used with national population estimates of age-, gender-, and race-specific blood pressure data to calculate average circumferential stress within demographic groups. The estimated circumferential stress at ages 55-74 years was about half that at ages 18-24 years because of a disproportionate increase of wall thickness relative to artery radius at older ages, violating the expected relationships described by the Laplace Law. Arteries with low circumferential stress (estimated at sites distant from atherosclerosis) had more necrotic atheromas than arteries with high stress. At sites with low stress and intimal thickening, smooth muscle cells (SMCs) were spread apart, thereby diminishing their density within both the intima and media. Thus, older arteries displayed both low circumferential stress and abundant matrix of low cellularity microscopically. Such changes might alter SMC-matrix interactions.

## INTRODUCTION

Separating arterial features of ordinary aging from those of atherosclerosis using various kinds of *in vivo* imaging techniques has been difficult in the past[1,2]. Postmortem histological specimens, when examined with attention to their limitations, can minimize some of these problems. Under physiologic conditions, the Laplace Law has been widely used by numerous investigators to explain the constancy of arterial wall thickness to lumen radius needed to maintain constant circumferential stress in the wall of the pressurized cylinder in a wide range of circumstances. Wolinskey and Glagov[3], for instance, measured wall-to-lumen ratios of abdominal aortas from a variety of mammalian species. From their data, assuming pressures of 100 mmHg, it is possible to calculate circumferential stress of 240,000 and 284,000 newtons per square meter (24.0 and 28.4 × $10^4$ N/$m^2$) for the averages of three mice and four bulls, respectively, a magnitude not significantly variable over a 20-fold range of aortic sizes. Other species (rat, cat, rabbit, dog, human, sheep, pig, and horse) all fell close to this same narrow range of values. In human arteries, however, data from postmortem specimens have repeatedly raised doubts about the constancy of radius-to-thickness ratios as a general property of aging atheroma-free arterial sites[4,5].

Much evidence suggests that the radius increases with age in human arteries, a process often ascribed to fractures of load-bearing elastin fibers in response to the fatiguing effect of tensile stress[6]. Wall thickness also increases with age[7,8], and the Laplace Law has sometimes been invoked to imply that the artery radius-to-wall thickness ratio should be held constant if the aging changes are the result of physiological adaptation[8]. This study was designed to examine the relationship of wall thickness, radius, estimated circumferential stress, and histologic features in right coronary arteries retrieved in a series of medico-legal autopsies.

So that atherosclerotic lesion thickening and remodeling would not cause biased calculations of circumferential stress, measurements were made at sites lacking any evidence of atherosclerosis.

## METHODS

### Source of Material

Coronary arteries were retrieved at autopsy from men and women of black and white ethnic groups aged 18–74 years in the Orleans Parish Coroner's Office in 2004-5 (n = 207). Only subjects with the right-dominant or balanced patterns of coronary circulation were retained. Left-dominant patterns, which affect about 10% of hearts, were excluded. A *basal* autopsy category was constructed using the subset of 182 subjects with deaths from violence, or from natural causes having no known relationship with hypertension or atherosclerosis. This basal group approximates a representative sample of the population[9] and these subjects were used for the analyses unless otherwise specified. The Institutional Review Board has declared this autopsy design as exempt from their review.

### Processing of Specimens

Each right coronary artery was opened longitudinally from the aortic root to the origin of the posterior descending branch. The heart was suspended by its own weight from a dowel in the tricuspid orifice as a way to distend the valve ring to its fullest extent. A piece of string was laid along the interior surface of the artery on its cardiac side, following the contours of the sometimes tortuous artery. The length of the string was used to estimate the *in vivo* artery length. Arteries were allowed to shrink by elastic recoil as they were dissected from the heart, then cut into segments, and each segment was flattened by compression with a sponge during formalin fixation. This process usually abolished tortuosity. Each artery's fixed length was the sum of its fixed segments' lengths. In exceptionally rigid tortuous arteries, the fixed segments retained a small arch leftward or rightward after flattening. Since the measured excised length was a straight line across the curves, the excised length in these few especially stiff specimens may have been underestimated slightly. This discrepancy is treated as a small component of residual error.

### Retraction Measure (Stiffness Index)

The length of each fixed artery can be expressed as a proportion of the string length, f = retracted length/h, where h is the *in situ* (string) length. This ratio, f, can be used as a stiffness index because it measures the relative failure of the excised specimen to retract. Part of this retraction is a small size change occurring during fixation (1–3%[10]), which is expected to vary little between specimens. The inverse ratio, 1/f, can be used to correct for retraction and shrinkage to estimate the *in vivo* artery measures. Heart weights were abstracted directly from autopsy protocols.

## Circumference Measurement

After arteries were flattened by pinning onto a cork board, measurements of luminal circumference were made at seven equidistant sites (>1-cm separations to assure independence). Luminal circumference was measured at the seven sites using a digital caliper accurate to 0.01 mm, and the mean radius was calculated for the specimen. If an assigned site on the artery was excessively thickened and stiffened by plaques, then a nearby site was substituted. In a few specimens, no substitute site could be found at one or more of the seven sites, and these few specimens were discarded. The coefficient of variability of these measurements averaged 11.5% in the arteries later found histologically to contain necrotic atheromas, and 13.7% ($p = 0.03$) in those without atheromas, indicating that, overall, a few widely scattered plaques or poststenotic dilatations did not produce greater unevenness of measured circumference.

## Slide Preparation

Longitudinally oriented samples of the formalin-fixed segments were embedded in paraffin on edge to allow lengthwise sectioning perpendicular to the luminal surface. Sections of 6-µm thickness were stained with hematoxylin and eosin (H&E). Shrinkage from the formalin-fixed state during section preparation averaged 17.2% (SEM = 0.96) in a sample of 20 specimens representing the full range of observed circumferential stresses, which is close to published values[10,11], and this factor was used to adjust histological measurements for this artifactual shrinkage. The correlation of this shrinkage with circumferential stress was $r = -0.05$ ($p = 0.84$), with stiffness $r = 0.032$ ($p = 0.89$), with age $r = 0.022$ ($p = 0.92$), and with presence of atheroma $t = 0.14$ ($p = 0.89$), showing that these properties of the arteries had minimal influence on the amount of shrinkage during paraffin embedding.

## Evaluation of Slides

Thicknesses of intima and media were measured with an eyepiece micrometer at nine equally spaced positions along the coronary sample (approximately 1-cm separations, sufficient to assure independence), taking the intima plus media to define total wall thickness, excluding adventitia. Counts of smooth muscle cell (SMC) nuclei were taken in an area defined by an eyepiece reticle to span a distance of 100 µm along the artery, and across the full thicknesses of intima and media. If sites had necrotic atheroma[4] or fatty streak elements[12], then they were excluded and nuclear counts were taken from nearby locations, so it is unlikely that nuclear counts contained significant numbers of immigrant leukocytes from the blood[13]. Whether aging may alter nuclear size, shape, and orientation is a topic for future quantitative evaluation, but subjectively, change in nuclear morphology does not appear to be a major contributor to the conspicuous expansion of distances between nuclei.

## Calculation of Circumferential Stress

Circumferential stress, s, was based on the simplified formula from the Laplace Law, $s = mr/t$, where the symbols m, r, and t denote mean arterial pressure (m), luminal radius (r), and wall thickness (t), respectively, as they apply to the living artery. The mean arterial pressure determines the SI units of measure for circumferential stress, 133.3 $N/m^2$ for each mmHg.

## Estimations of *In Vivo* Arterial Measures for Calculating Circumferential Stress

The *in situ* radius was estimated as r = circ/2πf, where circ is the observed mean circumference of the retracted specimen and f is defined above. This formula assumes that the longitudinal retraction is a reasonable estimate of the circumferential retraction, and this assumption will be examined later in the Discussion section. Using lower case letters hereafter for *in situ* dimensions, *in situ* wall thickness, t, which is needed to calculate circumferential stress, can be estimated as follows. Artery wall volume is calculated as the difference between the volumes of the two cylinders described by the outer wall and the lumen. So, for the retracted specimen, volume = length × π $(\text{radius} + \text{thickness})^2$ – length × π $(\text{radius})^2$. Treating as negligible the small effects of formalin fixation, this observed volume is then equated to the *in situ* volume, $\pi(r+t)^2h - \pi r^2h$ = retracted volume, in a manner similar to wall area calculations[14], from which t is readily extracted. Observations needed to determine stress in the longitudinal direction were not attempted here[15].

## Estimating Mean Arterial Pressure

Since mean arterial pressure (MAP) is not available in this data set and varies with age, we used published values of age-, gender-, and (black vs. white) race-specific diastolic and systolic blood pressures from the NHANES II[16] national survey data to estimate age-, race-, and gender-specific mean arterial pressures, m = MAP = (Systolic + 2Diastolic)/3. The survey estimate of MAP for each age, gender, and race group was substituted for the unknown value in each individual; these estimated values are thought to be reasonable approximations for calculating group averages of circumferential stress. Circumferential stress calculations were repeated after substituting systolic for mean blood pressure, because it is unknown whether wall architecture is responsive to mean or systolic pressures.

## Reproducibility of Measurements

The coefficient of variation (CV) for triplicate measurements of the *in situ* (string) lengths was calculated in16 unselected consecutive hearts and ranged from 0 to 6%, with a median of 1.7%. Circumference measured at multiple sites along the lengths of 207 arteries had CV ranging from 2.6 to 23.9%, with a median of 9%, while wall thickness CV ranged from 7.7 to 69.7%, with a median of 21.5%. Errors in the calculated circumferential stress are clearly dominated by within-specimen biological variation, rather than method error.

## Statistical Methods

Analyses all employ widely used methods offered in the SAS package of computer programs (SAS Institute, Cary, NC). Two-way analysis of variance (ANOVA) between genders and across five levels of age, applied to basal cases, was carried out with PROC GLM so that continuous covariates could be introduced. Heart weight was evaluated as a potential confounder in the derivation of estimated circumferential stress in this way. To derive a cutoff point for discriminating the existence of atheromas within coronary arteries, logistic regression evaluated 124 subjects, ages 35–74 years, with all causes of death; age-specific odds ratios were calculated from the 2 by 2 contingency tables using this cutoff point in all age groups.

## RESULTS

The findings in all tables derive from 136 men and 46 women who were 18–74 years of age, who had basal causes of death, and included blacks and whites. There were more men than women at all ages, with the greatest discrepancy in numbers being for the ages of 18–34 and 55–74. Occasional inconsistencies between men and women may relate to this deficiency in numbers. The full 207 specimens are used only in a scatter plot.

### Artery *In Situ* Length and Stiffness Index

After adjusting for insignificant racial differences, both age and gender were significantly associated with tethered artery length overall (Table 1). The tethered length was significantly longer at older ages among men, but age did not reach statistical significance among women where the sample size was smaller, and no significant interaction was found between age and gender ($p = 0.42$). Overall, adjusting for age and race, arteries in men were about 6% longer than arteries in women. In general, arteries retracted after removal from the heart and fixation even at the oldest ages (Table 1, stiffness index), but retraction was least at the oldest ages so that the excised length represented a large proportion of the *in situ* length. The loss of retraction in women was about twice the loss found in men over the observed age range, and a statistically significant age-gender interaction was found ($p = 0.01$).

**TABLE 1**
**Age-Specific Means* of Selected Gross Anatomic Characteristics of the Right Coronary Artery in Autopsy Cases with Basal Causes of Death**

| Age (Years) | Stiffness Index† (Ratio Units) | | Tethered (*In Situ*) Length (cm) | | | Number of Cases | |
|---|---|---|---|---|---|---|---|
| | Men | Women | Men | Women | All‡ | Men | Women |
| 18–24 | 0.800A | 0.752A | 10.6A | 10.4A | 10.5A | 30 | 8 |
| 25–34 | 0.830A | 0.755A | 11.2AB | 9.7A | 10.5AB | 37 | 6 |
| 35–44 | 0.841AB | 0.869B | 12.1BC | 11.0A | 11.6BC | 21 | 10 |
| 45–50 | 0.943C | 0.882B | 11.8BC | 11.6A | 11.7BC | 21 | 15 |
| 55–74 | 0.902BC | 0.955B | 12.4C | 11.9A | 12.2C | 27 | 7 |
| Mean ± SD‡ | 0.863 ± 0.078 | 0.841 ± 0.08 | 11.6 ± 1.4 | 10.9 ± 1.5 | 11.4 ± 1.4 | 136 | 46 |

* Means derive from generalized linear modeling, adjusting for black vs. white racial group as a covariate. Numbers of cases are applicable also to Tables 2–5.

† Excised length/tethered length.

‡ Age- and gender-specific means (± standard deviation) are adjusted for race; age-specific means for tethered length are additionally adjusted for gender.

*Note*: Tests of the main effects of age group on stiffness index ($p < 0.001$) and on tethered length ($p = 0.01$) are significant, while the main effect of gender is significant on tethered length ($p < 0.001$), but not on stiffness ($p = 0.13$); age by gender interactions were significant for stiffness ($p = 0.01$), but not for tethered length ($p = 0.42$). Post hoc tests: a pair of means within a column that fail to share a symbol A, B, C, or D differ significantly at $p < 0.05$ by Tukey HSD test.

## Artery *In Situ* Radius

The estimated *in situ* arterial radius (Table 2) also varied significantly with age for men ($p = 0.002$), but not for women ($p = 0.90$), which called for omission of women from segment-specific analysis. The association between age and radius was similar for all three arterial segments among men (age by segment interaction $p = 0.75$), although paired comparisons did not reach significance by Tukey tests in the distal segment. The significant decrease in arterial radius from the proximal to distal segments was modest as expected. Men tended to have larger radii than women at older, but not at younger, ages; however, the age-gender interaction was not significant ($p = 0.26$).

**TABLE 2**
**Age-Specific Means* of the Estimated *In Situ* Radii for Right Coronary Artery, Overall (n = 182) and by Gender, and for Longitudinal Segments among Men (n = 136) in Cases with Basal Causes of Death†**

| Age (Years) | Radius (mm); Men Only | | | Radius (mm) | | |
|---|---|---|---|---|---|---|
| | Right Coronary Artery Segment | | | | | |
| | Proximal | Middle | Distal | Men | Women | All |
| 18–24 | 1.47A | 1.34A | 1.23A | 1.36A | 1.41A | 1.39A |
| 25–34 | 1.58AB | 1.42AB | 1.27A | 1.43AB | 1.46A | 1.45AB |
| 35–44 | 1.69BC | 1.54ABC | 1.43A | 1.55AB | 1.37A | 1.46AB |
| 45–54 | 1.63AB | 1.58BC | 1.43A | 1.54AB | 1.40A | 1.47AB |
| 55–74 | 1.75C | 1.64C | 1.38A | 1.60B | 1.44A | 1.52B |
| Mean ± SD | 1.60 ± 0.26 | 1.48 ± 0.26 | 1.32 ± 0.26 | 1.50 ± 0.24 | 1.41 ± 0.25 | 1.47 ± 0.24 |

* See footnote in Table 1.

† Age- and segment-specific means (± standard deviation) are adjusted for race. Age- and gender-specific means are adjusted for race; age-specific means for men and women combined are adjusted for race and gender.

*Note*: Main effect of age in men are significant for proximal ($p = 0.002$), middle ($p < 0.001$), and distal ($p = 0.009$) segments, and the age by segment interactions are not significant ($p = 0.75$). Pooled segment effects of age were significant for men ($p = 0.002$), but not for women ($p = 0.90$); gender effect was significant ($p = 0.03$), but the interaction of age with gender was not significant ($p = 0.26$). Post hoc tests as in Table 1.

## Wall Thickness

Because the increments for wall thickness, for intimal percentage, and the wall-to-radius ratio were similar in men and women after adjusting for race ($p > 0.11$ for interactions with gender), no gender-specific results are shown (Table 3). The thicknesses of the total wall, intima, and media, adjusted for retraction to approximate *in situ* values, all varied significantly with age ($p < 0.001$). All measures tended to be thicker at older ages after adjusting for race and gender (each $p < 0.001$, Table 3). The increment for the intima was larger than for the media. The ratio of wall (intima plus media) thickness to radius did not vary significantly by gender ($p = 0.11$), but did vary significantly with age ($p < 0.001$), being larger in the two older groups relative to the three younger ages. In fact, the ratio in the oldest age group was twice that of the youngest age group. The age-adjusted partial correlation of wall thickness with stiffness was r = 0.56.

**TABLE 3**
**Age-Specific Means* of Coronary Artery Intimal, Medial, and Total Wall Thicknesses Adjusted for Retraction, % of the Wall that is Intima, and Thickness/Radius Ratio (t/r), Derived from 182 Autopsy Cases with Basal Causes of Death**

| Age (Years) | Thickness (mm) | | | Intima (%) | t/r (Ratio Units) |
|---|---|---|---|---|---|
| | Intima | Media | Total | | |
| 18–24 | 0.087A | 0.123A | 0.211A | 39.6A | 15.3A |
| 25–34 | 0.109A | 0.141A | 0.250AB | 43.0AB | 17.5A |
| 35–44 | 0.144B | 0.157A | 0.301B | 45.6BC | 21.1A |
| 45–54 | 0.211C | 0.196B | 0.408A | 50.5CD | 28.0B |
| 55–74 | 0.269C | 0.203B | 0.472C | 54.2D | 31.4B |
| Men, Mean ± SD† | 0.175 ± 0.089 | 0.180 ± 0.055 | 0.355 ± 0.117 | 46.0 ± 11.1 | 23.8 ± 8.2 |
| Women, Mean ± SD† | 0.153 ± 0.092 | 0.148 ± 0.056 | 0.301 ± 0.121 | 47.1 ± 11.5 | 21.5 ± 8.5 |

* See footnote in Table 1.

† Age-specific means are adjusted for race and gender; gender-specific means (± standard deviation) are adjusted for race and age.

*Note*: Tests of main effects of age on intima, media, total wall thickness, intima %, and t/r are all significant ($p < 0.001$ in all instances). Main effects by gender are significant for media ($p < 0.001$) and for total wall ($p < 0.01$), but not for intima ($p = 0.18$), for intima % ($p = 0.59$), or for t/r ($p = 0.11$). All interactions of gender by age are insignificant ($p > 0.11$).

## Circumferential Stress

The MAP abstracted from the NHANES II source increased by 17% for men and 24% for women from ages 18–24 to 55–74 years (Table 4). Circumferential stress, calculated using these blood pressure values with the estimates of the *in situ* arterial measures, varied significantly with age ($p < 0.001$, Table 4). However, instead of increasing with age as would be expected with the rise in pressure, circumferential stress declined by 41.9% from the youngest to the oldest ages ($p < 0.001$, Table 4). The lower stress at older ages was because the wall thickness increased disproportionately compared to the radius from youth to old age, and therefore overwhelmed the small countervailing effect of MAP in the calculations. Gender was neither a confounder ($p = 0.31$) nor an effect modifier of the age relationship to circumferential stress ($p = 0.27$ for age-gender interactions). Repeating the circumferential stress estimations using systolic pressures showed an absolute decline from 11.3 to 6.8 $N/m^2 \times 10^4$ (39.8%), indicating no serious disagreement with the proportionate decline obtained using mean pressures. For individual arteries, wall thickness declined in proportion to radius along the arterial length, so that within age ranges, the averages of circumferential stress were always constant along the arterial length of both genders (data not shown).

## Relationships to Necrotic Atheroma

The presence of at least one microscopic necrotic atheroma somewhere in the specimen increased from 1.6% of the youngest subjects to 75.0% of the oldest subjects when combining men and women and adjusting for race (Table 4). Circumferential stress measured throughout the nonatheromatous arterial regions tended to be lower in arteries that displayed atheroma elsewhere in the specimen than in those with no atheroma (Fig. 1, vertical line plots the following equation with W = 0 as explained next). A logistic regression equation was computed for using stress to determine a cutoff point for separating the specimens with atheroma from those without atheroma: $W = 3.88 - 0.657 \times \text{Stress}$.

**TABLE 4**
**Age-Specific Means* of Circumferential Stress and Prevalences of Atheroma in 182 Autopsy Cases with Basal Causes of Death, and Mean Blood Pressure from a Survey Source**

| Age (Years) | Circumferential Stress ($10^4$ N/$m^2$) | | | Atheroma Prevalence† (%) | | | Mean BP‡ (mmHg) | |
|---|---|---|---|---|---|---|---|---|
| | Men§ | Women§ | All§ | Men§ | Women§ | All§ | Men | Women |
| 18–24 | 7.9BC | 9.4D | 8.6C | 4.6A | 1.4A | 1.6A | 91.0 | 84.6 |
| 25–34 | 7.1C | 8.4CD | 7.7BC | 17.6A | 16.4A | 17.0AB | 95.6 | 87.8 |
| 35–44 | 7.3AB | 6.6BC | 6.9B | 27.7AB | 38.8AB | 33.2BC | 99.2 | 94.4 |
| 45–54 | 4.8A | 5.9AB | 5.3A | 51.5BC | 39.4AB | 45.5CD | 102.6 | 102.4 |
| 55–74 | 5.5A | 4.6A | 5.0A | 66.6C | 83.5B | 75.0D | 106.4 | 105.0 |
| Mean ± SD§ | 6.5 ± 2.6 | 7.0 ± 2.6 | | 33.6 ± 42.1 | 35.3 ± 43.5 | | | |

* See footnote in Table 1.

† Percentage of cases displaying at least one instance of necrotic atheroma in the specimen.

‡ Mean blood pressure = (Systolic + 2Diastolic)/3, using age-, race-, and gender-specific data from a survey source (NHANES II[16]).

§ Age by gender-specific means (± standard deviation) is adjusted for race, overall means additionally adjusted for gender.

*Note*: Main effects of age on stress are significant ($p < 0.001$), but for gender are not ($p = 0.27$). Main effects of age on atheroma are significant ($p < 0.001$), but for gender are not ($p = 0.81$). All interactions of gender with age are insignificant ($p > 0.27$).

When W > 0, stress < 5.9, and atheroma is declared likely to be present. This logistic regression equation with stress measured in units of its standard deviation becomes W = 3.88 – 1.38 × Stress with OR = 3.99 (95% CI = 2.30 to 6.94). The equation yields 29% false-negative and 25% false-positive predictions in the ages 35–74 years using this 5.9 cutoff point. Race, gender, and heart weight were not significant when included into the equation. Age yielded a small positive association with atheroma ($p = 0.02$) if added to the equation, but allowing this age effect to enter changed the coefficient for stress only to –0.567 (–14%) and the false diagnoses to 28 and 23%, respectively, judged to be negligibly small. Therefore, age was omitted for simplicity. This equation was derived from the 124 men and women of ages 35–74 years having all causes of death, and was tested among the 83 subjects in the age range 18–34 years in Fig. 1. In the figure, odds ratios (ORs) for age groups were calculated from the respective 2 by 2 contingency tables.

## SMC Numbers

Within a longitudinally oriented segment of arterial wall spanning a distance of 100 μm, and including intima plus media, the number of SMC nuclei varied significantly with age for intima, media, and total wall thickness ($p < 0.001$ for each), with ages over 44 years generally having more nuclei than in youth (Table 5). While fewer nuclei were found for women compared to men ($p < 0.001$ for intima, media, and overall), the association of number of nuclei with age was similar for men and women ($p > 0.12$ for all age-gender interactions). The thicknesses of intima, media, and total wall per SMC nucleus also were associated significantly with age ($p < 0.001$ for each), tending to be higher at older ages (Table 5), but were not associated with gender ($p > 0.46$ for each test), and gender did not modify the age effect (all age-gender interactions $p$ values > 0.12).

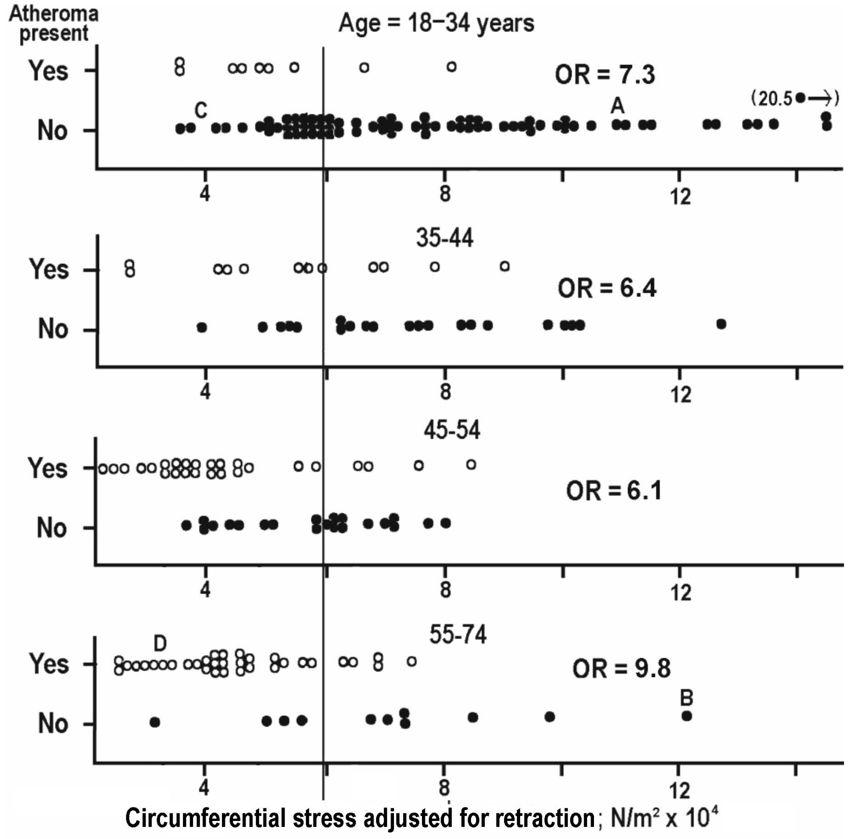

**FIGURE 1.** Vertical line at abscissa 5.9 plots the equation with W = 0, a cutoff point separating Yes from No specimens. ORs are for Yes/No classification. An outlier is noted by 20.5 ⇗. Letters A–D agree with Fig. 2.

## Heart Weight

Pearson partial correlations between heart weight and other measures were examined, adjusting for age, race, and sex, omitting coronary heart disease. Heart weight was significantly correlated with arterial radius (r = 0.415, $p$ <0.001) and thickness (r = 0.201, $p$ = 0.007), but not their ratio, t/r (r < 0.01, $p$ = 0.97), or circumferential stress (r < 0.01, $p$ = 0.99). These findings reflect the fact that radius and thickness vary with heart weight in proportion to each other so that their ratio remains constant at all heart sizes (data not shown), and circumferential stress therefore also remains constant.

## Photographic Examples

Representative examples of young and old subjects with low and high calculated circumferential stresses are compared in Fig. 2. An example of a commonplace youthful subject with high stress (frame A) shows closely spaced SMCs and relatively little matrix that has an open and loose appearance in both intima and media. An uncommon elderly subject who displayed high stress (frame B) shows fewer SMCs, which are more widely spaced in a denser collagenous matrix than that seen in youth. The wall thickness is greater in frame A than B because the youthful artery has recoiled upon excision (excised/*in situ* length = f = 0.705) more than the elderly one (f = 0.791), and thus the two have a comparable circumferential stress after greater downward adjustment of thickness for the effect of retraction. An example of an unusual youthful subject who displayed low levels of circumferential stress (frame C) shows a thick wall with increased matrix per

**TABLE 5**
**Age-Specific Means* of Selected Microscopic Features for the Right Coronary Artery in 182 Autopsy Cases with Basal Causes of Death**

| Age (Years) | SMC Numbers† | | | Thickness per SMC (µm/SMC) | | |
|---|---|---|---|---|---|---|
| | Intima | Media | Total Wall | Intima | Media | Total Wall |
| 18–24 | 10.1A | 41.1A | 51.2A | 8.6A | 3.0A | 4.1A |
| 25–34 | 11.0A | 41.7AB | 52.8A | 10.3A | 3.4AB | 4.8A |
| 35–44 | 13.2AB | 49.1AB | 62.4AB | 10.9AB | 3.3AB | 4.9A |
| 45–54 | 17.2BC | 55.0B | 72.1B | 12.6BC | 3.6BC | 5.7B |
| 55–74 | 18.0C | 52.2AB | 70.0B | 14.8C | 4.0C | 6.8B |
| Men‡, Mean ± SD | 15.1 ± 6.1 | 52.3 ± 13.6 | 67.4 ± 16.4 | 11.2 ± 3.5 | 3.4 ± 0.6 | 5.2 ± 1.2 |
| Women‡, Mean ± SD | 12.7 ± 6.3 | 43.3 ± 2.1 | 56.0 ± 17.0 | 11.7 ± 3.6 | 3.4 ± 0.7 | 5.2 ± 1.3 |

* See footnote in Table 1.

† SMC nuclei numbers in a 100-µm wide band through the artery wall.

‡ Age by gender-specific means is adjusted for race, and overall means additionally adjusted for gender.

*Note*: Main effects of age and gender are significant for SMC numbers in the intima, media, and total wall ($p < 0.001$ in all instances). For thickness per SMC, $p < 0.001$ for age differences of intima, media, and total wall, but for gender differences $p = 0.47$ for intima, $p = 0.82$ for media, and $p = 0.77$ for total wall. Interactions of age by gender are all insignificant ($p > 0.12$).

SMC (51% greater in frame C than A), which spreads the cells apart. Comparing the commonplace elderly example (frame D) with the unusual youthful example (frame C) shows the wall thickening to be more in the intima of the elderly and less in the media. The two examples of low stress arteries exhibited almost no retraction after dissection from the heart (f = 0.923 and 0.952 for arteries shown in frames C and D, respectively). So the *in situ* wall thicknesses estimated by adjusting for retraction are little changed from those actually observed in the photographs.

## DISCUSSION

Coronary arteries retract upon removal from the heart. This retraction can produce estimates of the *in vivo* dimensions that are falsely low for radius and falsely high for wall thickness, yielding deceptive values for circumferential stress. Thus, associations of the stress estimates with other variables, such as age, gender, heart size, etc., will be biased, unless corrections can be applied to adjust for these effects of retraction. This investigation introduces a novel method to correct for coronary artery retraction in postmortem specimens. The method uses longitudinal retraction, which can easily be measured, as a substitute for the desired quantity of radial retraction, which is not easily attainable. The method simplistically assumes that postmortem radial retraction can be treated as proportional to longitudinal retraction. This assumption is not rejected after the following review of relevant literature, and appears to be a reasonable approximation. In our exploratory analyses, we found a number of interesting associations that provide working hypotheses for further consideration.

Some readily available published tabulations of *in vivo* angiogram determinations provide data for estimating the radius in plaque-free proximal right coronary arteries (RCA) of middle-aged and elderly men; these are 1.83 mm[17], 1.70 mm[18], 1.60/1.65/1.65 mm[19], 1.81 mm[20], 1.54/1.29 mm[21], 1.80 mm[22], and 1.65 mm[23]. The one report[24] that classifies the arteries in terms of dominant circulatory patterns reported RCA radii of 1.50 mm for a balanced pattern (the most common), 1.90 mm for small right-dominant pattern, 1.95 mm for right-dominant pattern, and 1.40 mm for left-dominant pattern (the

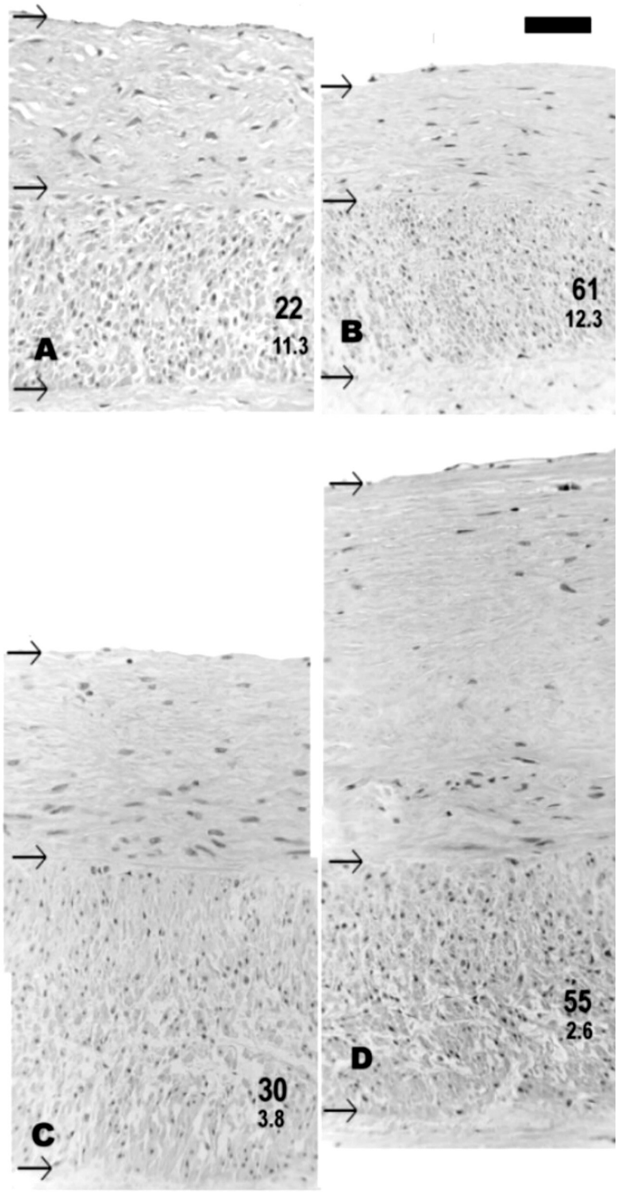

**FIGURE 2.** Longitudinal coronary planes compare young (A and C) with old (B and D) subjects having high (A and B) and low (C and D) circumferential stresses. Numerals give age (years) over circumferential stress (N/m$^2$ × 10$^4$). Arrows mark luminal surface, intima-media boundary, and media-adventitia boundary. Letters A–D agree with Fig. 1. Bar = 50 μm; H&E.

least common). The average of all of the cogent estimates (eliminating left-dominant) is 1.66 mm, which compares with 1.63 and 1.75 mm for proximal segment in men of ages 45–74 years in Table 2. Although a number of limitations in our study call for caution, these Table 2 findings appear to be in good agreement with the *in vivo* radiological data. Studies making comparable measurements of inflation-fixed postmortem specimens found radii of 1.96 mm[19] and 1.87 mm[25], a 15 and 11%, respectively, discrepancy from the *in vivo* average. In assessing this discrepancy, Schulte-Altedorneburg et al.[26] measured dimensions of common carotid arteries by ultrasound imaging in patients with late-stage diseases and then repeated the measurements on the carotid arteries retrieved at autopsy. The radii in distention-fixed postmortem specimens averaged 9.5% greater than those *in vivo* and the excess was attributed to overdistention of the inflation-fixed flaccid postmortem specimens. Bank et al.[27] used ultrasound imaging to measure the luminal cross-sectional area of brachial artery before and after administering sublingual nitroglycerine, and found a 17% increase of radius in the paralyzed arteries, which seems consistent with the values of 15 and 9.5% just reviewed.

Bank et al.[27] further determined the arterial luminal area at zero pressures induced by inflation of a cuff on the upper arm. Their estimates of diminished radius from normal baseline were 71 and 84% obtained by two different imaging techniques, which compares with 75.2 to 86.9% for dissected retraction in the younger subjects of Table 1. Hence, the circumferential retraction observed by Bank et al. *in vivo* is similar to the longitudinal retraction in our postmortem specimens, in keeping with our original assumption.

Williams et al.[28] used sensor-tipped catheters in the left branches of the coronary artery in 20 subjects of average age 56.6 years, with plaques only in the right branch to determine wall stress at systolic pressures averaging 139.0 mmHg. Their tabulated data allow calculation of the 95% confidence interval (CI) of 8.97 to 10.53 (mean 9.75) × $10^5$ dynes/cm$^2$. For comparison with this, the age-, gender-, and race-adjusted values in the age range 40–69 years in the present study were used to calculate circumferential stress using the value of 139 mmHg for MAP in the formula. This approach neglects the wall thinning that accompanies the higher pressure and is expected to yield a low estimate for circumferential stress. The stress estimates obtained in this way were 6.58 to 7.46 (mean 7.03) × $10^5$ dynes/cm$^2$. The small discrepancy in these comparisons does not seem to indicate any clear reason to reject our method for estimating postmortem circumferential retraction given the numerous variations in methodology between the two studies.

In the U.S. population, blood pressure rises with age and this contributes to the circumferential stress imposed upon aging arteries. To the extent that the NHANES II survey data of MAP fail to precisely parallel the specific, but unknown, pressures in the subjects of this study, some error will occur in the estimated stress values. However, the nearly twofold rise in thickness-to-radius ratio over the range of ages in Table 3 would require an accompanying increase of MAP to 210 mmHg (e.g., 270/180 mmHg) for ages 55–74 years to sustain constant circumferential stress. Clearly, whatever errors may have been introduced by the substitution of survey data for the missing MAP values cannot account for the magnitude of departure from Laplace expectations observed here.

Among women of ages 18–34 years in this study (data not shown), the calculated circumferential stress in the right coronary artery ranged from 14.68 to 5.57 ×$10^4$ N/m$^2$, a 2.6-fold range (subsample of Fig. 1). This range seems not too unreasonable for a biological variable that is held in physiological homeostasis, and these women might be viewed as representing what is "normal" (cf. Fig. 2A). For men of this age group, however, circumferential stress ranges from 14.53 to 3.55 ×$10^4$ N/m$^2$ (excluding one outlier), a 4.1-fold range, which seems somewhat harder to reconcile with the concept of homeostatic regulation. This observation raises the possibility that some of these young men may already be acquiring pathological changes of aging[29].

Carallo et al.[30] summarize a widely held view that, "In humans, aging is accompanied by a subversion of arterial wall, which involves splitting and fractures of elastic fibers and increased collagen fibers and intercellular matrix. The vessel becomes larger and stiffer, probably because the relative function of elastic laminae is lost . . ." Najjar et al.[31] review the substantial body of evidence that calls for this condition to be viewed as a diagnostic rubric. A name for this condition, "senile ectasia", is credited to Aschov (1924) by Mitchell and Schwartz[32] (cf. Fig. 2D, SMCs are pressed widely apart by the profound increase in densely packed collagenous matrix).

The Laplace Law, s = mr/t (cf. "calculating circumferential stress" in methods), when applied to the data reported here on aging coronary arteries, finds s to vary substantially across age groups, thereby violating the constancy to be expected from findings among normal youthful specimens. However, within age groups, stress remains constant between genders, over the range of heart sizes, and along the lengths of arteries. Hence the arterial response to the forces described by the law appears to behave as expected within age ranges, but to depart from expectation between age groups. A way to summarize these findings is to introduce a modification to the usual formula: s = kmr/t. If k = 1 is chosen for ages 18–24 years, then k diminishes progressively to k = 0.58 at ages 55–74 years in these data (5.0 ÷ 8.6 = 58% from Table 4). It is not circumferential stress, s, that remains constant with age, but s/k, the modified stress term. This study was not designed to investigate possible cell-matrix interactions that might contribute to anatomic changes and reduced circumferential stress. These interactions are objects of intense inquiry and many details are known[2,15,33,34,35,36,37]. One may speculate that k may herald some changes of cell-matrix interactions, perhaps because of replacement of elastica by collagen for sustaining tension in the aging artery. Anisotropy between arterial layers will be of considerable importance in these considerations[17]. The present study offers no data to comment upon possible molecular mechanisms of this disturbance, but this newly described clue might open novel directions for pursuing these matters.

Increasing age and low circumferential stress were found to be associated with a number of histologic characteristics. An increased prevalence of necrotic atheromas and greater wall thickness per SMC nucleus are two of the interesting findings. Because our study is cross-sectional and had limited risk factors available for study, we cannot determine the sequence of anatomic change or whether some unstudied factor that increases with age caused both the anatomic changes and the low circumferential stress.

Of the many limitations in this exploratory study, three in particular call for some selected comment: (1) stress is distributed heterogeneously across the arterial wall, (2) stress values range widely in the youngest ages, and (3) circumferential retraction is heterogeneous across the arterial wall.

1. With the intimal and medial walls displaying variations between them, and from place to place within them, it seems clear that circumferential stress does not fall equally on all structural elements. It seems plausible to suggest that the subset of elements that sustain the distending forces may be experiencing tensions like those in youth and that much of the wall is to some degree flaccid. However, such heterogeneity fails to explain the existence of those redundant flaccid elements, which are not attempting to adjust their architecture appropriately to the sensed tensions. Indeed, relative to the youthful state, the aged arterial wall is experiencing a degree of laxity, either in whole or in part, and the same dilemma calls for resolution in either case.
2. The youngest women display a 2.6-fold range of measured values for stress. This could be construed as excessive for a variable held in homeostasis. In the men, there is clear evidence that the ectasia of aging is already setting in to some subjects by ages 20–24 years and begins in the media to extend later to intima[29], as illustrated by the example in Fig. 1C. These unusual subjects could account for decreases into the lower range of stress if they also occur in women, a matter that is unclear. The few instances of excessively high stress could be exceptional because their actual blood pressure was lower than the average value applied to them here, or because of exceptional extremes in unrecognized methodological failures. These influences would be further exaggerated if pulse pressure or pulse rate, highly variable quantities, could also impact wall thickness, effects that would cancel out in the group averages. Yet it is hard to presume a range of less than twofold and this seems unreasonably large. Whereas the Laplace expectations can be useful in comparisons of group averages, they are often uncertain when applied to individuals. This discrepancy can be resolved only by further studies.
3. When the dissected artery is opened longitudinally, it sometimes happens that the media shows greater circumferential retraction than the intima. This is observed when the artery inverts to form an inside-out cylinder, sometimes almost to completion. The phenomenon is generally absent before age 30–35 years and again after age 45–50 years. In most specimens, the phenomenon does not occur and therefore poses no problem. When it does occur, the artery is pinned in a way that flattens the intima, leaving the media not fully flat; in these few specimens, the measured circumference is that of the intima (inner circumference) and not the media. This does not necessary translate into greater stress in the *in vivo* distension of media, because even when distended, the flexible media could remain relative lax compared to the intima. Referring to the discussion of topic 1 above, these arteries are presumably among those where a degree of laxity affects part but not all of the arterial thickness.

The architectural changes evolving in aging coronary arteries at sites distant from atherosclerosis are well known in at least broad outline. These changes, however, are widely referred to as "adaptive thickening" and are thought to constitute a form of "normal" intima, often attributed to physiological adaptations in keeping with the Laplace relationships, and having no importance for the evolution of atherosclerotic plaques[12]. In contrast to this conventional wisdom, the present report builds upon a growing body of information that suggests that these architectural changes may have a central place in the evolution of plaques[1,2,3,4,5,6,7,8,31,38,39,40].

## ACKNOWLEDGMENTS

This work was conducted without grant funding. Citations are all to published sources with no outside consultation.
